# AKT2 suppresses pro-survival autophagy triggered by DNA double-strand breaks in colorectal cancer cells

**DOI:** 10.1038/cddis.2017.418

**Published:** 2017-08-24

**Authors:** Nina Seiwert, Carina Neitzel, Svenja Stroh, Teresa Frisan, Marc Audebert, Mahmoud Toulany, Bernd Kaina, Jörg Fahrer

**Affiliations:** 1 Department of Toxicology, University Medical Center Mainz, Mainz, Germany; 2 Department of Cell and Molecular Biology, Karolinska Institute, Stockholm, Sweden; 3 Toxalim Research Centre in Food Toxicology, Université de Toulouse, INRA-UMR1331, ENVT, INP-Purpan, UPS, Toulouse Cedex 3, France; 4 Division of Radiobiology and Molecular Environmental Research, Department of Radiation Oncology, University of Tuebingen, Tuebingen, Germany; 5 German Cancer Consortium (DKTK), partner site Tuebingen, and German Cancer Research Center (DKFZ), Heidelberg, Germany

## Abstract

DNA double-strand breaks (DSBs) are critical DNA lesions, which threaten genome stability and cell survival. DSBs are directly induced by ionizing radiation (IR) and radiomimetic agents, including the cytolethal distending toxin (CDT). This bacterial genotoxin harbors a unique DNase-I-like endonuclease activity. Here we studied the role of DSBs induced by CDT and IR as a trigger of autophagy, which is a cellular degradation process involved in cell homeostasis, genome protection and cancer. The regulatory mechanisms of DSB-induced autophagy were analyzed, focusing on the ATM-p53-mediated DNA damage response and AKT signaling in colorectal cancer cells. We show that treatment of cells with CDT or IR increased the levels of the autophagy marker LC3B-II. Consistently, an enhanced formation of autophagosomes and a decrease of the autophagy substrate p62 were observed. Both CDT and IR concomitantly suppressed mTOR signaling and stimulated the autophagic flux. DSBs were demonstrated as the primary trigger of autophagy using a DNase I-defective CDT mutant, which neither induced DSBs nor autophagy. Genetic abrogation of p53 and inhibition of ATM signaling impaired the autophagic flux as revealed by LC3B-II accumulation and reduced formation of autophagic vesicles. Blocking of DSB-induced apoptotic cell death by the pan-caspase inhibitor Z-VAD stimulated autophagy. In line with this, pharmacological inhibition of autophagy increased cell death, while ATG5 knockdown did not affect cell death after DSB induction. Interestingly, both IR and CDT caused AKT activation, which repressed DSB-triggered autophagy independent of the cellular DNA-PK status. Further knockdown and pharmacological inhibitor experiments provided evidence that the negative autophagy regulation was largely attributable to AKT2. Finally, we show that upregulation of CDT-induced autophagy upon AKT inhibition resulted in lower apoptosis and increased cell viability. Collectively, the findings demonstrate that DSBs trigger pro-survival autophagy in an ATM- and p53-dependent manner, which is curtailed by AKT2 signaling.

Autophagy is a highly conserved cellular process, in which cytoplasmic components are engulfed in vesicles, termed autophagosomes, and delivered to lysosomes for degradation.^1^ The resulting low-molecular breakdown products are fuelled into the *de novo* synthesis of cellular macromolecules or serve as an energy source, both of which are essential under stress conditions.^2^ Autophagy, therefore, has a crucial role both in the maintenance of cell homeostasis and recycling of damaged organelles as well as misfolded proteins.^3^ It is also engaged in the protection of genome stability.^4^ Consistent with this notion, autophagy was reported to exert tumor-suppressor functions at early stages of carcinogenesis, as loss of the autophagy regulator *beclin-1* or deletion of *atg5* resulted in increased tumorigenesis.^5, 6^ On the other hand, autophagy induction by nutrient deprivation and hypoxia sustains tumor cell viability by providing metabolic substrates and promotes tumor progression.^7, 8^

It was previously shown that autophagy is activated in response to reactive oxygen species (ROS). This effect was mediated by stimulation of the LKB1/AMPK/TSC2 axis and involved the cytoplasmic activation of ATM.^9^ ATM is an integral component of the DNA damage response (DDR), which is activated by DNA double-strand breaks (DSBs) in the nucleus. DSBs are very critical DNA lesions, which threaten both cell survival and genome integrity.^10^ DSBs can be directly generated by ionizing radiation (IR), radiomimetic anticancer drugs and bacterial protein toxins referred to as ‘cytolethal distending toxins’ (CDTs).^11, 12, 13^ Furthermore, DSBs can arise indirectly due to the collapse of stalled replication forks at sites of DNA damage, for example bulky DNA adducts.^14^ DSBs can result in chromosomal aberrations, which are causally linked to cancer formation,^15^ and are a potent trigger of apoptotic cell death.^16^

IR is a well-established DSB inducer, which is used to study DSB-related cellular pathways.^17^ However, IR generates not only DSBs but also a plethora of other DNA lesions, including DNA single-strand breaks (SSBs) and oxidative base modifications.^18^ Some of these lesions can be converted to DSBs during DNA replication.^18^ IR further triggers membrane signaling and modifies membrane constituents by lipid peroxidation.^19, 20^ In contrast, CDT produced by Gram-negative bacteria causes exclusively DNA strand breaks owing to its intrinsic DNase I-like endonuclease activity.^21^ The toxin enters mammalian cells via dynamin-dependent endocytosis followed by its retrograde transport into the nucleus.^21^ At high doses, CDT generates DSBs via introduction of overlapping SSBs in close proximity at the opposite strands, while at low doses it induces mainly SSBs that are converted into DSBs in a replication-dependent manner.^22, 23^

In view of the important role of autophagy in genome protection and cancer, we set out to dissect the DSB-induced autophagy and the underlying regulatory mechanisms with a focus on AKT signaling and p53 in colorectal cancer (CRC) cells.

## Results

### The radiomimetic toxin CDT and IR trigger autophagy

**Figure 1:**
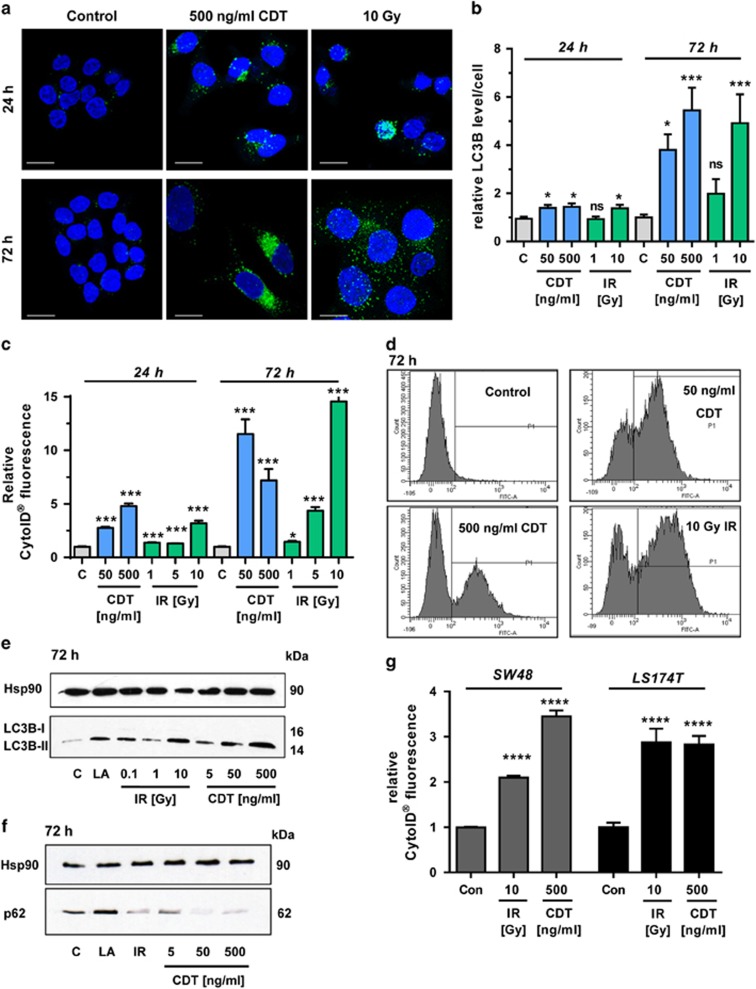
Time- and dose-dependent induction of autophagy following DNA DSBs. (**a** and **b**) Time- and dose-dependent induction of the autophagy marker LC3B. HCT116 cells were treated with CDT or exposed to *γ*-irradiation and incubated for up to 72 h. Cells were then fixed and stained for LC3B (green). Nuclei were visualized by TO-PRO-3 (blue). Images were recorded by confocal microscopy and LC3B intensity was quantified by ImageJ (*n*=3, 8–10 sections per sample). ***P<0.001, *P<0.05, NS: not significant, *versus* untreated controls. Scale bar represents 20 *μ*m. (**c**) Time- and dose-dependent induction of autophagosomes. HCT116 cells were treated as described above and harvested after 24 or 72 h. Autophagic vesicles were stained with the CytoID Autophagy Detection Kit and measured by flow cytometry. *n*=3; NS: not significant; ***P<0.001, *P<0.05, *versus* control cells. (**d**) Representative histograms of CytoID staining. (**e** and **f**) Impact of CDT and IR on LC3B-II and p62 levels after 72 h. Cells were treated with CDT or IR in a dose-dependent manner and harvested after 72 h. Samples were analyzed by SDS-PAGE and western blotting as indicated. Hsp90 was visualized as loading control. LA was used as positive control. (**g**) Determination of autophagosomes in SW48 and LS174T cells upon DSB induction. Cells were treated as indicated and harvested after 24 h. Autophagic vesicles were stained with the CytoID Autophagy Detection Kit and measured by flow cytometry. *n*=3; ****P<0.0001, *versus* control cells

First, HCT116 CRC cells were treated with CDT or exposed to IR. Both caused an increase in LC3B-positive vesicles already after 24 h (Figures 1a and b), which further accumulated after 72 h (Figures 1a and b). The increased level of LC3B staining was already detectable in cells exposed to 50 ng/ml CDT for 24 h (Figure 1b and Supplementary Figure 1A), which further augmented after 72 h (Figure 1b and Supplementary Figure 1B). The formation of autophagic vesicles was then monitored by CytoID staining, showing a strong increase upon CDT and IR treatment, with a maximum after 72 h (Figures 1c and d; Supplementary Figure 1C). High doses of IR and CDT caused an increase of the autophagosome marker LC3B-II after 24 h (Supplementary Figure 1D), which was much more pronounced after 72 h, reaching the levels of the positive control *α*-lipoic acid (LA)^24^ (Figure 1e). Concomitantly, the autophagy receptor p62, which is degraded in the late stages of autophagy, was analyzed. The p62 level was reduced at the highest CDT dose after 24 h and almost undetectable after CDT treatment for 72 h, suggesting a stimulation of the autophagic flux (Figure 1f and Supplementary Figure 1E). Irradiation of cells with 10 Gy elicited a transient increase of p62 followed by a drop below control levels after 72 h (Figure 1f and Supplementary Figure 1E). The findings were confirmed in SW48 CRC cells, in which both CDT and IR caused an accumulation of LC3B-II. Concurrently, both stimuli resulted in the degradation of p62, which was already detectable at a dose of 50 ng/ml CDT (Supplementary Figure 2A). In line with these findings, IR or CDT treatment increased autophagosome formation in SW48 and in LS174T CRC cells (Figure 1g and Supplementary Figure 2B). Importantly, DSB-induced autophagy was not only restricted to CRC cells but also occurred in HeLa S3 cervix carcinoma cells (Supplementary Figures 2C and D). It should also be noted that both CDT and IR caused a G2–M arrest after 24 h (Supplementary Figure 3C), which is in accordance with previous reports.^25, 26^ Importantly, the apoptotic subG1 population was very low, thereby excluding early cytotoxic effects. Taken together, both the radiomimetic bacterial toxin CDT and IR induce autophagy in a dose- and time-dependent manner in different CRC cell lines.

### DSB formation is required for autophagy induction and mTOR repression

**Figure 2:**
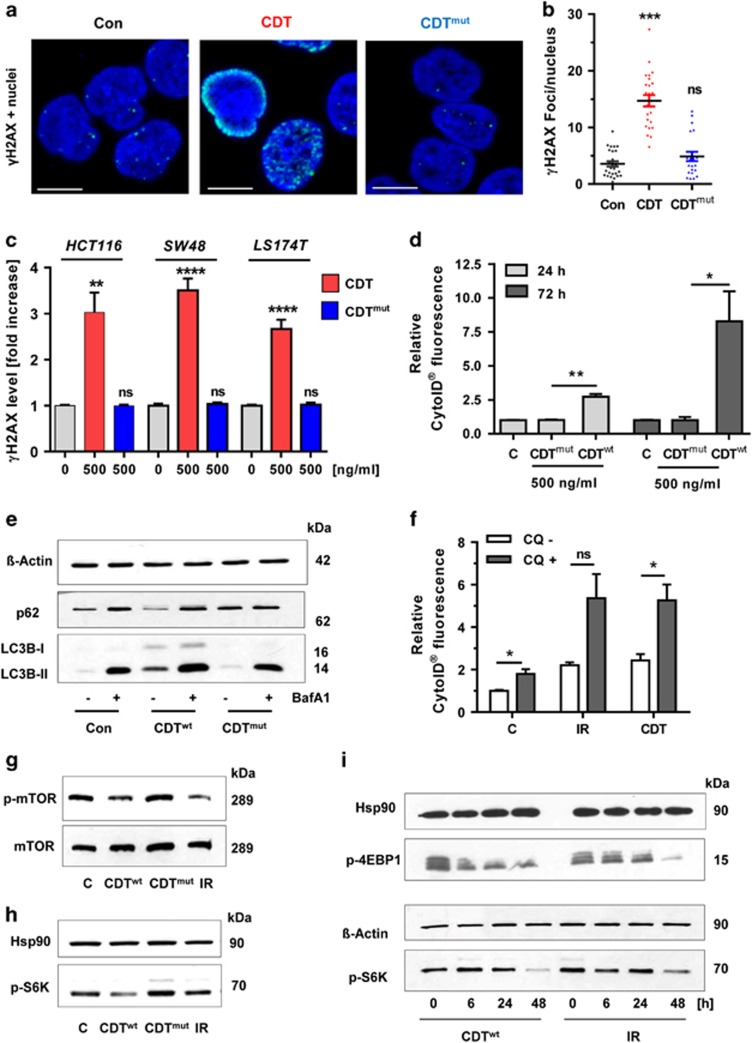
DSB formation is required for autophagy induction and mTOR repression. (**a**) Induction of *γ*-H2AX foci by wild-type CDT and mutant CDT (CDT^mut^) lacking DNase activity. HCT116 cells were treated with CDT or CDT^mut^ for 24 h (500 ng/ml each), stained with a *γ*-H2AX antibody and subjected to confocal microscopy. Representative images are shown. *γ*-H2AX is depicted in green and nuclei are blue. Scale bar represents 10 *μ*m. (**b**) Quantitative evaluation of *γ*-H2AX foci in HCT116 cells as shown in panel (**a**). The number of *γ*-H2AX foci per nucleus were determined by the ImageJ software and evaluated with GraphPad Prism 5.0 (>50 cells per experiment; *n*=3); NS: not significant. ***P<0.001, *versus* control. (**c**) ICW analysis of *γ*-H2AX formation in different CRC cell lines treated with wild-type or mutant CDT. Cells were exposed to CDT or its DNase I-defective mutant (CDT^mut^) as described above. After 24 h, cells were processed for ICW analysis and *γ*-H2AX level was determined as compared with untreated control cells (*n*=5). NS: not significant. **P<0.01, ****P<0.0001. (**d**) Analysis of autophagosome formation in HCT116 cells upon treatment with DNase I-deficient CDT. Cells were incubated with CDT or CDT^mut^ (500 ng/ml each) for 24 or 72 h. Induction of autophagosomes was revealed by the CytoID Autophagy Detection Kit and flow cytometry. *n*=3; **P<0.01, *P<0.05. Bars in light gray: 24 h; bars in dark gray: 72 h. (**e**) Monitoring of autophagic flux. HCT116 cells were incubated with CDT or CDT^mut^ for 21 h, then supplemented or not with the autophagy inhibitor Baf A1 (10 nM) and incubated for another 3 h. After 24 h, cells were harvested and analyzed by SDS-PAGE followed by western blotting detection of LC3B and p62. *β*-Actin served as loading control. (**f**) HCT116 cells were treated with CDT or exposed to IR. After 30 h, the autophagy inhibitor CQ (10 *μ*M) was added and cells were further incubated for 18 h. Cells were harvested after 48 h and autophagy induction was measured using the CytoID Autophagy Detection Kit (*n*=3). NS: not significant. *P<0.05. (**g**) Impact of DSB induction on mTOR signaling. HCT116 cells were treated with CDT, CDT^mut^ or IR and harvested after 48 h. Samples were then separated by SDS-PAGE and analyzed by western blotting detection of mTOR and phospho-mTOR. (**h**) Detection of the mTOR substrate phospho-p70-S6K in cells treated as described above for 24 h. Hsp90 served as loading control. (**i**) Time-dependent repression of mTOR signaling by CDT and IR. HCT116 cells were exposed to CDT or IR and incubated for up to 48 h. Samples were then analyzed as described above followed by detection of the mTOR substrate phospho-p70 S6K and phospho-4E-BP1

To detail the contribution of DSBs to autophagy induction, a DNase I-defective CDT mutant (CDT^mut^) was used.^27^ Confocal microscopy revealed up to 15 *γ*-H2AX foci per cell after treatment with wild-type CDT, while the mutant showed a similar number of *γ*-H2AX foci as untreated control cells (Figures 2a and b). These results were confirmed in all tested CRC cell lines using in-cell western (ICW) analysis (Figure 2c and Supplementary Figure 3A). In contrast to wild-type CDT, treatment of HCT116 cells with CDT^mut^ did not affect the viability (Supplementary Figure 3B). The effects of CDT^mut^ on autophagy were tested using CytoID staining, showing lack of autophagosome formation (Figure 2d). In line with this finding, the DNase-defective mutant neither provoked degradation of p62 nor induced accumulation of LC3B-II (Figure 2e). Using the autophagy inhibitor bafilomycin A1 (Baf A1), an increase of both p62 and LC3B-II was observed in cells incubated with wild-type CDT, revealing stimulation of the autophagic flux (Figure 2e). This was corroborated in autophagosome measurements using the autophagy inhibitor chloroquine (CQ) (Figure 2f and Supplementary Figure 4A). Next, the impact of DSB induction on mTOR as central negative autophagy regulator was assessed. Treatment of cells with wild-type CDT or IR repressed mTOR phosphorylation on Ser-2448 (Figure 2g). Accordingly, the phospho-modification of p70 S6 kinase (S6K), a well-known downstream target of mTOR,^28^ was decreased upon DSB induction (Figure 2h). Importantly, cells treated with DNase-defective CDT displayed no alterations in mTOR signaling. Additional time course studies showed the strongest repression of mTOR signaling after 48 h, with a reduced phosphorylation of p70 S6K and 4E-BP1 (Figure 2i), which is another mTOR substrate.^28^ In summary, these results strongly suggest that DSB induction is essential to trigger autophagy with concomitant mTOR repression.

### Role of the ATM-p53-mediated DDR in DSB-induced autophagy

**Figure 3:**
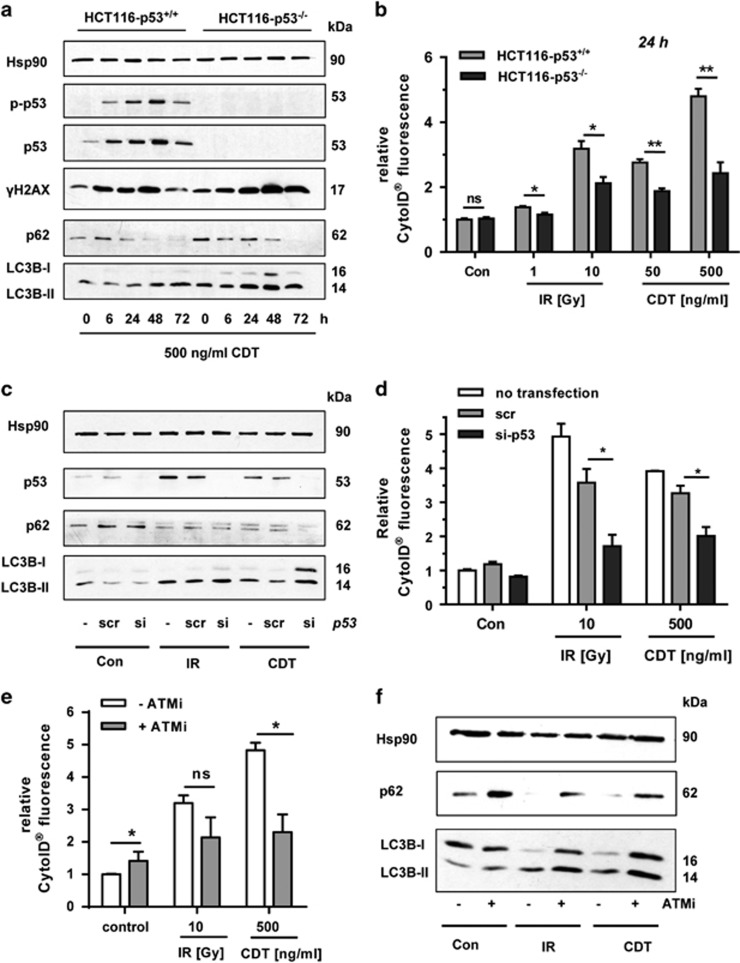
Impact of p53 and ATM on DSB-induced autophagy. (**a**) Time course of DNA damage response signaling and autophagy induction in isogenic p53-proficient and -deficient HCT116 cells. HCT116-p53^+/+^ and HCT116-p53^−/−^ cells were challenged with CDT for up to 72 h. Cell lysates were then subjected to SDS-PAGE followed by western blotting detection as indicated. Hsp90 served as loading control. (**b**) Determination of autophagic vesicles in HCT116-p53^+/+^ and HCT116-p53^−/−^ cells following DSB induction. Cells were irradiated or exposed to CDT as indicated and incubated for 24 h. Autophagosomes were stained with CytoID reagent and monitored by flow cytometry. (*n*=3); NS: not significant. *P<0.05, **P<0.01. (**c**) siRNA-mediated knockdown of p53 and accumulation of LC3B. HCT116 cells were transiently transfected with scrambled (scr) or p53-specific siRNA. Twenty-four hours after transfection, cells were treated with 500 ng/ml CDT or exposed to IR (10 Gy) and incubated for 48 h. Whole-cell extracts were analyzed by immunoblot detection using antibodies against p62 and LC3B. Hsp90 served as loading control, whereas p53 was visualized to confirm efficient knockdown. (**d**) Autophagosome formation upon siRNA-mediated depletion of p53. HCT116-p53^+/+^ cells were transfected with p53 siRNA or scrRNA and treated as described above. Autophagic vesicles were detected by CytoID staining and flow cytometry. (*n*=3); NS: not significant. *P<0.05. (**e**) Effect of ATM inhibition on DSB-induced autophagosome formation. Cells were pretreated with the pharmacological ATM inhibitor KU-55933 (ATMi) and then exposed to either CDT or IR. After 48 h, autophagosomes were stained as described before (*n*=3); NS: not significant. *P<0.05. (**f**) Effect of ATM on DSB-dependent LC3B accumulation. Cells were treated as mentioned above and subjected to SDS-PAGE followed by western blotting analysis

The tumor-suppressor protein p53 is activated by diverse stimuli, including DNA damage,^29^ and modulates autophagy.^30^ To assess its function in DSB-triggered autophagy, we used isogenic p53-proficient and -deficient HCT116 cells. CDT treatment caused an accumulation of p53 in wild-type HCT116 cells, coinciding with its phosphorylation at Ser-15, whereas no p53 was detectable in p53-negative HCT116 cells (Figure 3a). CDT further induced *γ*-H2AX in both cell lines with a maximum after 48 h (Figure 3a). LC3B-II levels also increased time-dependently, although this was more pronounced in p53-deficient cells (Figure 3a). Accordingly, the autophagy substrate p62 decreased after CDT exposure (Figure 3a). The formation of autophagic vesicles was then assessed, showing a significant reduction in HCT116-p53^−/−^ cells both after IR and CDT treatment (Figure 3b and Supplementary Figure 4B). p53 was then transiently downregulated in HCT116-p53^+/+^ cells (Figure 3c). Both CDT- and IR-treated cells displayed an accumulation of p53 after 48 h, except for the cells with p53 knockdown. As observed before, CDT and IR stimulated LC3B-II formation and concomitant p62 degradation. However, cells with downregulated p53 exhibited higher levels of LC3B-II following IR and CDT incubation (Figure 3c), which reconciles the findings obtained above (Figure 3a). The formation of autophagic vesicles was compromised in cells with transient p53 knockdown (Figure 3d), which is also consistent with the data obtained with the p53-deficient cells. These results indicate that DSB-mediated p53 activation is necessary for a complete autophagic flux.

p53 is stabilized via its phosphorylation at Ser-15, which is catalyzed by the apical DDR kinases, such as ATM,^31^ and is induced by CDT or IR.^23^ To address the contribution of ATM, a pharmacological inhibitor (ATMi) was used. Cells were exposed to either IR of CDT in the presence or absence of ATMi and autophagosome formation was monitored. ATMi strongly decreased the number of autophagic vesicles in cells treated with CDT and, to a lesser extent, also in cells exposed to IR (Figure 3e). Cells co-treated with ATMi showed a reduced degradation of p62 and increased LC3B levels both under basal conditions and upon DNA damage induction, also suggesting an impaired autophagic flux in the absence of ATM signaling (Figure 3f). In conclusion, our results provide evidence that p53 and its upstream activator ATM are involved in DSB-triggered autophagy by promoting the autophagic flux.

### Interplay of autophagy and cell death upon DSB induction

**Figure 4:**
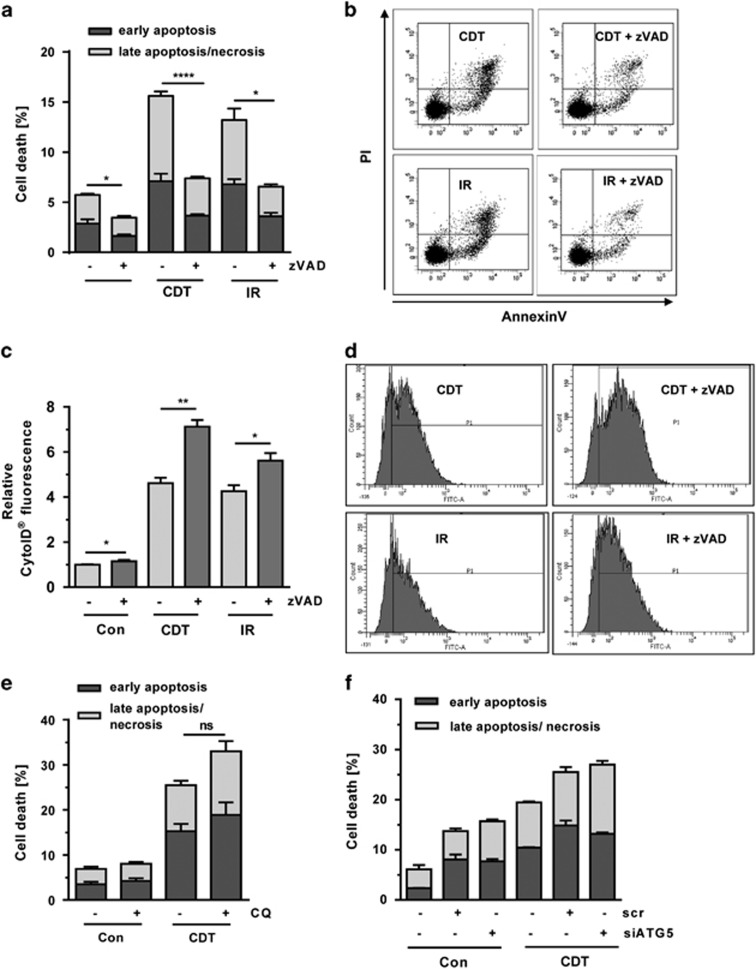
Interplay between DSB-induced autophagy and cell death. (**a**) Cell death induction by CDT and IR and involvement of caspases. HCT116 cells were treated with CDT (500 ng/ml) or exposed to IR (10 Gy). After 20 h, cells were supplemented with the pan-caspase inhibitor zVAD (20 *μ*M) and incubated for another 28 h. Cell death was finally assessed by Annexin V/PI staining and flow cytometry (*n*=3); ***P<0.001, *P<0.05. (**b**) Representative dot plots of Annexin V/PI measurements. (**c**) Impact of pan-caspase inhibition on DSB-induced autophagy. HT116 cells were treated as described above and processed for CytoID staining followed by flow cytometry (*n*=3); **P<0.01, *P<0.05. (**d**) Representative histograms of CytoID staining. (**e**) Chemical inhibition of autophagy and DSB-induced cell death. HCT116 cells were exposed to CDT as described above and supplemented with CQ (10 *μ*M). After 48 h, cells were harvested, stained with Annexin V/PI and analyzed by flow cytometry. (*n*=3); NS: not significant. (**f**) Impact of ATG5 knockdown on CDT-induced cell death. HCT116 cells were transfected with ATG5 siRNA or scrambled RNA. After 24 h, cells were exposed to CDT (500 ng/ml) and incubated for additional 48 h. Cell death induction was monitored by flow cytometry (*n*=3)

Previous reports have shown that CDT triggers caspase-dependent cellular demise.^23, 26^ In order to figure out whether autophagy is upstream or downstream of apoptosis, we used the pan-caspase inhibitor Z-VAD. HCT116 cells were exposed to CDT or IR in the absence or presence of Z-VAD and incubated for 48 h. Annexin V/PI staining revealed moderate levels of cell death induction upon CDT or IR treatment (Figures 4a and b). Pan-caspase inhibition almost completely rescued the cells (Figures 4a and b), suggesting that DSBs trigger predominantly caspase-dependent apoptosis. At the same time, CytoID staining was performed, showing an induction of autophagic vesicles in response to CDT and IR (Figures 4c and d). Interestingly, supplementation of Z-VAD to cells treated with CDT or IR significantly increased autophagosome formation (Figures 4c and d), arguing against the notion that autophagy acts downstream of apoptosis. To further address the role of autophagy in cell survival upon DSB induction, cells were treated with CDT and supplemented with the autophagy inhibitor CQ. CDT-induced cell death was further increased by CQ addition, which was, however, not statistically significant (Figure 4e). Finally, autophagy was genetically abrogated by small interfering RNA (siRNA)-mediated downregulation of ATG5 (Supplementary Figure 4C), which did not affect CDT-induced cell death (Figure 4f). Taken together, these findings provided evidence that autophagy acts rather upstream of apoptosis and not vice versa. In addition, the data indicate a pro-survival role of autophagy following DSB induction.

### AKT activation by DSBs restrains autophagy

**Figure 5:**
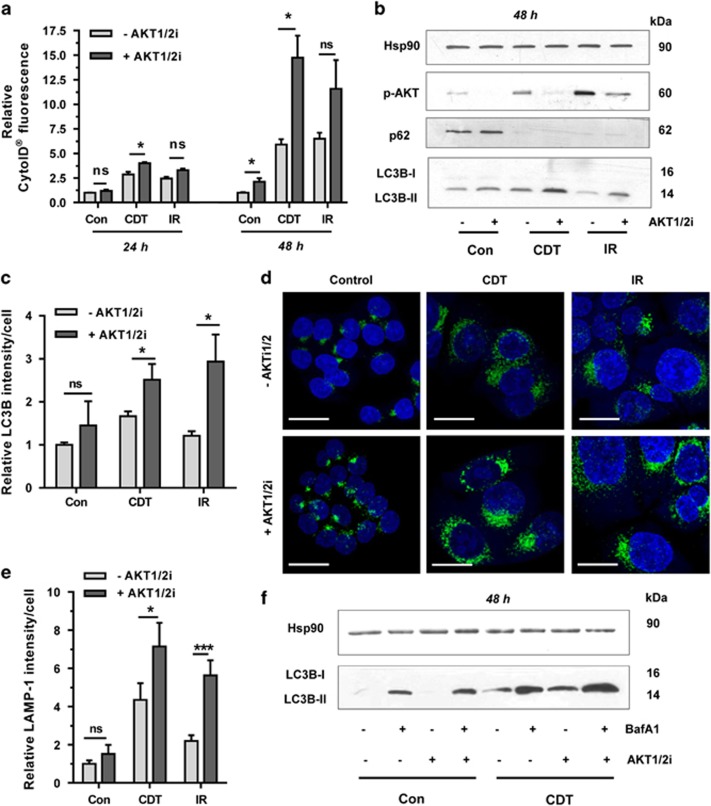
DSB-mediated AKT activation restrains concomitant autophagy induction. (**a**) Impact of AKT on DSB-induced autophagosome formation. HCT116 cells were treated with CDT (500 ng/ml) or irradiated (10 Gy) in the absence or presence of an AKT1- and AKT2-specific inhibitor (AKT1/2i; 1 *μ*M). Cells were harvested after 24 or 48 h and then processed for CytoID staining followed by flow cytometry (*n*=3). NS: not significant. *P<0.05. (**b**) Effect of AKT on DSB-induced LC3B accumulation and p62 degradation. Cells were treated as described above and analyzed by western blotting detection. Hsp90 served as loading control. (**c**) Impact of AKT on DSB-induced LC3B-positive vesicles. Cells were incubated for 48 h as mentioned above and LC3B-positive vesicles were determined by confocal immunofluorescence microscopy. (*n*=3) NS: not significant. *P<0.05. (**d**) LAMP-1 staining following DSB induction in the absence or presence of AKT signaling. Cells were incubated for 48 h as described, processed for LAMP-1 staining and analyzed by confocal microscopy. Representative images are shown. LAMP-1 is depicted in green and nuclei are shown in blue. Scale bar: 20 *μ*m. (**e**) Quantitative evaluation of LAMP-1 staining as shown in panel (**e**). LAMP-1 intensity was quantified by ImageJ (*n*⩾3, 8–10 sections per sample). ***P<0.001, *P<0.05, NS: not significant. (**f**) Impact of AKT inhibition on autophagic flux. HCT116 cells were incubated with CDT in the absence or presence of an AKT1- and AKT2-specific inhibitor (AKT1/2i; 1 *μ*M) for 45 h, then supplemented or not with the autophagy inhibitor Baf A1 (10 nM) and incubated for another 3 h. After 48 h, cells were harvested and subjected to SDS-PAGE followed by western blotting detection of LC3B. Hsp90 served as loading control

Both ATM and p53 were previously linked to protein kinase B/AKT,^32, 33^ a key player in the class I PI3K pathway.^34^ As AKT is engaged upon DSB formation, we investigated its role in DSB-induced autophagy. HCT116 cells were incubated with CDT or irradiated in the absence or presence of a pharmacological inhibitor specific for the AKT isoforms AKT1 and AKT2 (AKT1/2i). CytoID measurements revealed a considerable increase of autophagic vesicles provided that AKT1/2 was inhibited (Figure 5a; Supplementary Figures 5A and B). Western blotting analysis confirmed CDT- and IR-triggered phosphorylation of AKT at Ser-473, which was abrogated by AKT1/2 inhibition (Figure 5b and Supplementary Figure 6A). After 48 h of incubation, LC3B-II levels increased in cells co-treated with CDT or IR and AKT1/2i (Figure 5b), whereas no changes were detectable after 24 h. However, p62 bands were much weaker in the setting of AKT1/2 inhibition (Supplementary Figure 6A), which altogether suggests an upregulation of autophagy in the absence of AKT signaling. In line with these results, confocal microscopy showed an increased LC3B level and an upregulation of LAMP-1, a marker for autolysosomes, in cells treated with CDT or IR together with the AKT1/2 inhibitor (Figures 5c–e and Supplementary Figure 6B). Finally, we assessed autophagic flux in the presence of AKT1/2 inhibition using Baf A1. Both basal and CDT-induced formation of LC3B-II was further augmented by AKT1/2 and Baf A1 co-treatment as compared with the single treatments. This suggests an upregulation of the complete autophagic flux given that AKT signaling is abolished (Figure 5f). Consistent with this, a co-staining of LC3B and LysoTracker showed a further increase of both LC3B and LysoTracker-positive vesicles in the presence of AKT1/2 inhibition after DSB formation (Supplementary Figures 7A and B). These results lend further support to the notion that AKT signaling restrains the autophagic flux, which fits to the accelerated degradation of p62 after AKT inhibition.

**Figure 6:**
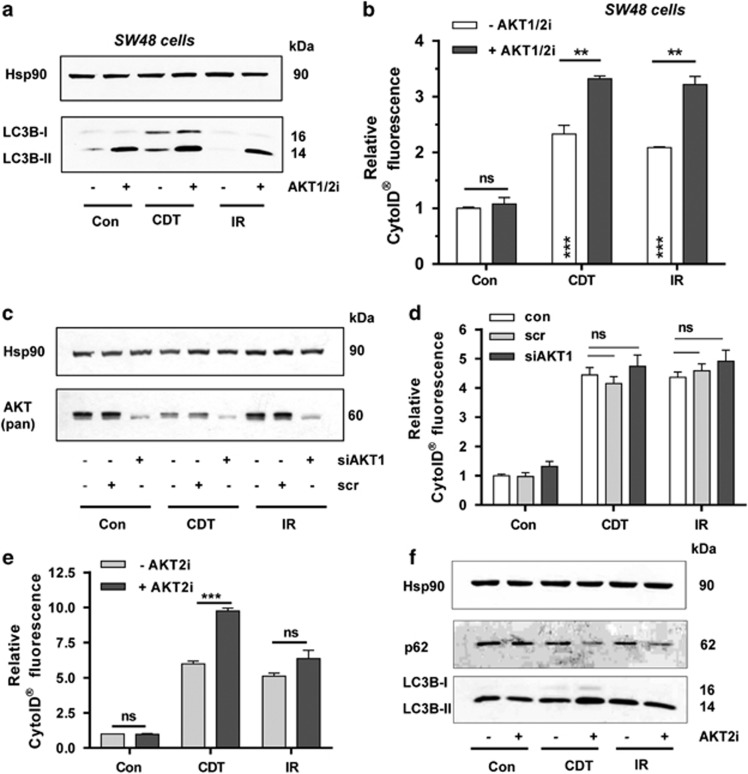
Isoform-dependent suppression of DSB-induced autophagy by AKT. (**a**) Impact of AKT on LC3B accumulation in SW48 cells. The cells were treated with CDT (500 ng/ml) or irradiated (10 Gy) in the absence or presence of an AKT1- and AKT2-specific inhibitor (AKT1/2i; 0.5 *μ*M). Cells were harvested after 48 h and subjected to western blotting analysis as indicated. Hsp90 served as loading control. (**b**) Induction of autophagosomes in SW48 cells in the presence of absence of AKT signaling. Cells were challenged as described above, processed for CytoID staining and then analyzed by flow cytometry. (*n*⩾3); ***P<0.001, **P<0.01, NS: not significant. (**c**) Transient siRNA-mediated knockdown of AKT1. HCT116 cells were transfected with AKT1 siRNA or scrambled siRNA. After 24 h, cells were exposed to CDT (500 ng/ml) or IR (10 Gy) and incubated for additional 48 h followed by western blotting analysis using a pan-AKT antibody. Hsp90 served as loading control. (**d**) AKT1 knockdown and DSB-induced autophagosomes. Cells were treated as described in panel (**c**) and autophagosome formation was monitored by CytoID staining and flow cytometry. (*n*=3). NS: not significant. (**e**) Pharmacological inhibition of AKT2 and formation of autophagic vesicles following DSB generation. HCT116 cells were treated with CDT (500 ng/ml) or exposed to IR (10 Gy) and incubated for 48 h in the absence or presence of an AKT2-specific inhibitor (AKT2i; 1 *μ*M). Autophagosome formation was measured using the CytoID Staining Kit and analyzed by flow cytometry. (*n*⩾3); ***P<0.001; NS: not significant. (**f**) AKT2 inhibition and DSB-induced LC3B accumulation and p62 degradation. Cells were treated as described in panel (**e**) and subjected to western blotting analysis for LC3B and p62. Hsp90 was detected as loading control

The negative regulation of autophagy by AKT signaling was then substantiated in SW48 CRC cells. A clear accumulation of LC3B-II was observed in SW48 cells upon CDT treatment, which was augmented by AKT inhibition (Figure 6a). With regard to IR, LC3B-II was only detectable with concomitant AKT inhibition and might indicate an enhanced degradation of LC3B upon irradiation. Determination of autophagic vacuoles demonstrated a strong stimulation of autophagy in SW48 cells if AKT1/2 is inhibited (Figure 6b). Taken together, the findings demonstrate that AKT activation suppresses the autophagic flux, which can be reversed by pharmacological AKT1/2 inhibition.

### AKT2 is responsible for the suppression of DSB-induced autophagy

In order to clarify which AKT isoform downregulates autophagy after DSB formation, AKT1 was depleted by siRNA knockdown in HCT116 cells followed by exposure to CDT or IR. Forty-eight hours after transfection, western blotting analysis showed efficient depletion of AKT1 (Figure 6c; Supplementary Figures 8A and B). The remaining faint band likely represents AKT1 or may stem from other AKT isoforms (AKT2 and/or AKT3). Interestingly, CDT treatment reduced the total AKT level as compared with untreated control cells (Figure 6c). The formation of autophagic vesicles was then determined by flow cytometry, however, without any effect in AKT1-downregulated cells (Figure 6d), pointing to AKT2 as crucial negative modulator. This was checked using a pharmacological inhibitor specific for AKT2. Intriguingly, cells treated with CDT or IR together with the AKT2 inhibitor recapitulated the effects observed following AKT1/2 inhibition (Figure 6e *versus* Figure 5a). This was substantiated by western blotting analysis, showing an enhanced degradation of p62 upon AKT2 inhibition in cells treated with either CDT or IR (Figure 6f). The LC3B-II level was potentiated in cells treated with CDT and AKT2 inhibitor but was slightly decreased in irradiated cells co-incubated with the inhibitor, which may reflect an advanced stage of autophagy and hence LC3B degradation. Altogether, the data show that AKT2 is the crucial isoform suppressing DSB-triggered autophagy.

### AKT inhibits pro-survival autophagy after DSB formation independent of DNA-PK

**Figure 7:**
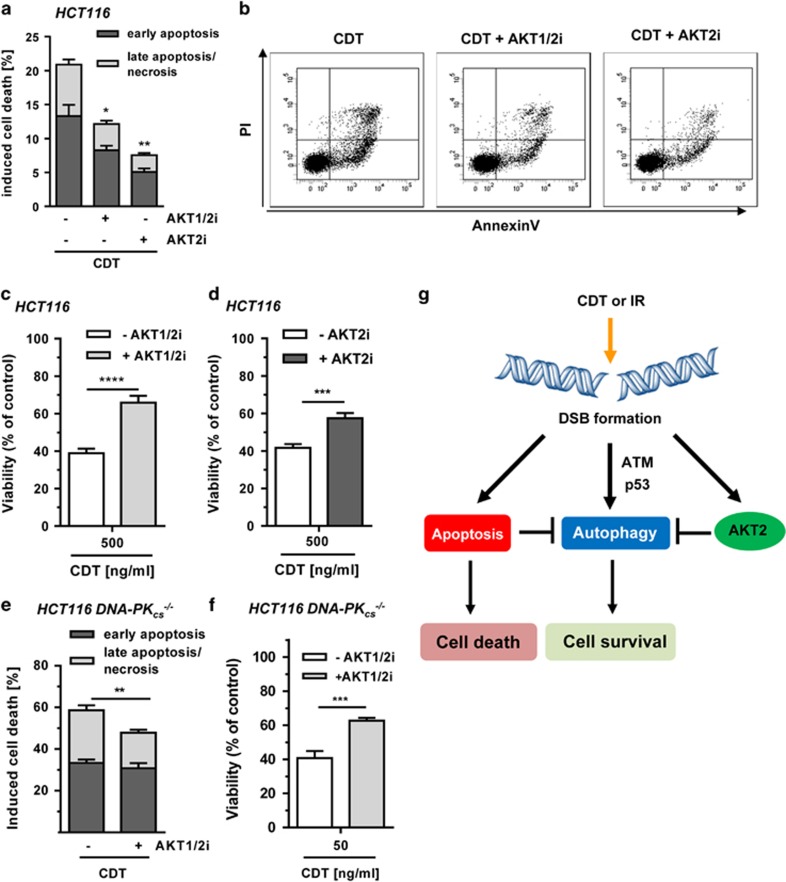
Impact of AKT on cell survival following DSB induction. (**a**) Role of AKT and autophagy in DSB-induced cell death. Cells were exposed to CDT (500 ng/ml) for 48 h with or without AKT1/2 inhibition (1 *μ*M) or AKT2 inhibition (1 *μ*M). Cells were harvested and stained with Annexin V/PI. Early apoptotic and late apoptotic/necrotic cells were then determined by flow cytometry. (*n*=3); **P<0.01, *P<0.05. (**b**) Representative dot plots of Annexin V/PI staining. (**c** and **d**) Influence of AKT on cell viability upon DSB generation. Cells were incubated with increasing doses of CDT for 72 h in the absence or presence of AKT1/2 (0.1 *μ*M) or AKT2 (5 *μ*M) inhibitor. Viability was then assessed using MTS assay. (triplicates, *n*=3); ****P<0.0001, ***P<0.001, (**e**) AKT inhibition and DSB-induced cell death in DNA-PK-deficient cells. Cells were treated and processed as described under panel (**a**). (*n*=3); **P<0.01. (**f**) Impact of AKT on viability of DNA-PK-deficient cells upon DSB induction. Cells were challenged and viability was measured as mentioned in panel (**b**). (triplicates, *n*=3); ***P<0.001. (**g**) Proposed model of DSB-triggered autophagy and its regulation by AKT. IR or CDT-mediated DSB formation stimulates the autophagic flux in an ATM- and p53-dependent manner. Concomitantly, DSB formation provokes caspase-dependent apoptotic cell death, which suppresses autophagy. DSB-triggered AKT signaling, mainly attributable to AKT2, curtails pro-survival autophagy independent of DNA-PK. Inhibition of AKT signaling boosts autophagy and thereby cell survival

In view of the negative regulation of autophagy by AKT, we wished to assess how AKT affects DSB-induced cell death. HCT116 cells were exposed to CDT in the presence or absence of the AKT1/2 inhibitor. CDT treatment on its own induced about 20% cell death, which was markedly reduced by co-incubation with the AKT1/2 inhibitor or the AKT2-specific inhibitor (Figures 7a and b). As another end point, viability was assessed 72 h following CDT treatment in HCT116 cells, revealing a strong decrease (Figure 7c). The CDT-mediated cytotoxicity was significantly attenuated by simultaneous AKT1/2 inhibition, which is consistent with the reduced levels of cell death (Figures 7a and b). The AKT2-specific inhibitor showed a similar effect and also increased viability upon CDT exposure (Figure 7d).

The PI3K-related kinase DNA-PK has previously been shown to phosphorylate AKT on Ser-473 following *γ*-irradiation.^35^ Thus, the contribution of DNA-PK_cs_ to AKT-mediated autophagy suppression was analyzed in isogenic DNA-PK_cs_-deficient HCT116 cells (Supplementary Figure 8C). DSB induction in HCT116-DNA-PK_cs_^−/−^ cells resulted in similar effects as in HCT116 wild-type cells, including AKT phosphorylation and autophagy induction (Supplementary Figures 8D and E). AKT inhibition further stimulated autophagy levels close to those observed in HCT116 wild-type cells. Annexin V/PI measurements revealed a strong cell death induction in DNA-PK_cs_-deficient cells, which was reduced by AKT1/2 inhibition (Figure 7e). This is comparable to the effect observed in DNA-PK_cs_-proficient, wild-type HCT116 cells (Figure 7a). Accordingly, pharmacological abrogation of AKT1/2 in cells exposed to CDT resulted in increased cell viability (Figure 7f). Taken together, inhibition of AKT signaling resulted in increased pro-survival autophagy with lower cell death and increased viability following CDT treatment. A model of DSB-induced autophagy is outlined in Figure 7g, showing the isoform-specific regulation by AKT and biological consequences.

## Discussion

Here we set out to characterize the role of a specific type of DNA damage, namely, DSBs, in autophagy induction and studied the underlying regulatory mechanisms. First, we demonstrate that both the radiomimetic bacterial toxin CDT and IR induce autophagy. This was not cell line specific but observed in several CRC cell lines, such as HCT116, SW48 and LS174T cells that express wild-type p53 and PTEN.^36^ Using autophagy inhibitors, we confirmed that DSB formation triggers the whole autophagic flux. This was completely dependent on DNA strand break induction as demonstrated with a DNase I-lacking CDT mutant. Intriguingly, DSB formation repressed mTOR signaling, which was in turn unaffected after CDT^mut^ exposure. DSB induction attenuated phosphorylation of mTOR at Ser-2448. This phospho-modification is catalyzed by either AKT^37^ or its downstream target p70-S6K in a positive feedback loop.^38^ Interestingly, we observed in the course of this study that both CDT and IR led to the activation of AKT as discussed below.

Next, the role of p53 in DSB-induced autophagy was addressed. Our results strongly suggest that p53 facilitates the autophagic flux and autophagosome maturation. This is in line with a previous report showing that loss of p53 decreases the autophagic flux with an accumulation of LC3 under nutrient deprivation.^39^ Furthermore, the DNA damage-regulated autophagy modulator DRAM was described to promote autophagy in a p53-dependent manner.^40^ Upstream of p53 is the DDR kinase ATM, which is activated by DSBs and subsequently orchestrates the DDR.^41^ Using a pharmacological ATM inhibitor, a pronounced decrease in autophagic vesicle formation and a concomitant accumulation of LC3B-II were revealed upon both CDT and IR, which parallels the findings obtained in the absence of p53. The observed effects are supported by an earlier study, showing an involvement of ATM in autophagy regulation upon *O*^6^-MeG-triggered DSBs.^42^ Furthermore, ROS have been shown to activate a cytoplasmic pool of ATM, which stimulates TSC2 via AMPK leading to the suppression of mTOR.^9^

Unrepaired DSBs are cytotoxic and provoke apoptotic cell death.^16, 23^ In order to integrate the observed autophagy in this context, the interplay between DSB-induced cell death and autophagy was studied. We showed that autophagy precedes apoptosis and is further increased if apoptotic cell death is blocked by pan-caspase inhibition, assigning DSB-induced autophagy a pro-survival role. The findings also highlight the well-known crosstalk between autophagy and apoptosis.^43^ Caspases were reported to cleave several ATG proteins, which thereby suppress autophagy and stimulate apoptosis.^44^ The observed pro-survival function of autophagy is supported by experiments with the autophagy inhibitor CQ that increased cell death. Transient knockdown of ATG5, required for autophagosome formation, showed no influence on DSB-induced cell death. This may be attributable to the fact that even very low levels of ATG5 are sufficient for normal function of autophagy.^45^

IR and CDT treatment induced phosphorylation of AKT on Ser-473, which is required for full AKT activation.^34^ We provided first evidence that pharmacological inhibition of AKT1 and AKT2 substantially increased DSB-mediated autophagy and stimulated the whole autophagic flux. Altogether, this reveals AKT as an important player of a negative feedback process in cells facing deleterious DSBs. AKT activation may thereby limit prolonged and excessive levels of autophagy in cells, which could trigger autophagic cell death or autosis.^46, 47^ Our data extend previous findings that AKT inhibits basal autophagy in prostate cancer and glioma cells.^48^ By a combination of genetic and chemical approaches, we identified AKT2 as the hitherto unknown AKT isoform curtailing autophagy upon DSB induction. In turn, AKT1 was recently described as an inhibitor of chaperone-mediated autophagy (CMA).^49^ Interestingly, the AKT1-mediated inhibition of CMA was abrogated by its upstream regulator PHLPP1, catalyzing AKT Ser-473 dephosphorylation.^49^

Finally, we provide evidence that inhibition of AKT1 and AKT2 supports viability in cells exposed to CDT. This was rather unexpected as AKT is a central part of the PI3K–AKT–mTOR pathway, which is frequently altered in cancer.^50^ In this context, it should be noted that HCT116 cells bear a H1047R mutation in the *PIK3CA* gene, which encodes the p110*α* catalytic subunit of PI3Ks.^51^ One explanation for the increased cell survival after AKT inhibition could be the concomitant induction of autophagy. AKT inhibition was previously shown to induce cytoprotective autophagy in ovarian cancer cells and increased CQ-induced cell demise.^52^ Our findings further support the notion that autophagy is a pro-survival pathway following CDT-induced DSB induction, which was also observed in irradiated cancer cells using autophagy inhibitors.^53^

In a last set of experiments, the role of DNA-PK in AKT-regulated autophagy was assessed. DNA-PK was reported as the kinase responsible for the phosphorylation of AKT on Ser-473 following induction of DSBs.^35^ However, our results indicate no differences with regard to AKT phosphorylation or autophagy induction. In line with these results, pharmacological abrogation of AKT also reduced cell death induction in DNA-PK-deficient cells. Here CDT elicited stronger cytotoxicity as compared with DNA-PK-proficient HCT116 cells, which is very likely attributable to the defects in NHEJ as shown earlier.^23^ It is conceivable that other apical DDR kinases compensate for DNA-PK and might catalyze the AKT phosphorylation in DNA-PK_cs_-deficient cells upon DNA damage.

Bacterial genotoxins have been implicated in the etiology of CRC. Pathogenic *E. coli* strains that express cyclomodulins, including CDT, were found at higher levels in colorectal tumor tissue than surrounding normal mucosa, correlating with CRC progression.^54^ In line with this observation, CDT-positive strains of *Helicobacter hepaticus* displayed improved colonization of the intestinal epithelium.^55^ Very recently, it has been demonstrated that DNA damage induction by typhoid toxin promotes long-term infection of the liver.^56^ It is further known that chronic exposure of cells to sublethal, low doses of CDT causes genetic instability and increases anchorage-independent growth,^57^ which was corroborated in normal human colonic epithelial cells carrying a defective APC tumor-suppressor gene.^58^ Thus, the CDT-induced activation of pro-survival pathways as shown here likely facilitates the survival of cells with acquired genetic damage and may therefore represent an increased risk to overcome the tumorigenesis barrier.

In conclusion, our study demonstrates that DSBs trigger autophagy in CRC cells dependent on p53 with concomitant downregulation of mTOR signaling. This pro-survival pathway is suppressed in an isoform-specific manner by AKT2. These findings bear significant implications not only for DNA damage-induced carcinogenesis but also for cancer radiotherapy.

## Materials and methods

### Materials

Baf A1, CQ and LA were purchased from Sigma (Deisenhofen, Germany). The AKT1/2-specific inhibitor AKTVIII was obtained from Merck Millipore (Darmstadt, Germany). The ATM inhibitor KU-55933, the AKT2-specific inhibitor CCT128930 and the pan-caspase inhibitor Z-VAD-FMK were from Selleck Chemicals (Houston, TX, USA).

### Cell lines and culture conditions

The two isogenic human CRC cell lines HCT116-p53^+/+^ and HCT116-p53^−/−^ were generously provided by Dr. Bert Vogelstein (John Hopkins University, Baltimore, MD, USA) and were originally obtained from the Core Cell Center (John Hopkins University) in 2012. Isogenic DNA-PK_cs_-deficient HCT116 cells (HCT116-DNA-PK_cs_^−/−^) were a kind gift of Dr. Eric Hendrickson (University of Minnesota, Minneapolis, MN, USA) in 2015. Cells were re-authenticated by p53 and DNA-PK_cs_ immunoblotting and by their characteristic differential response to 5-FU (WT *versus* p53^−/−^) and radiomimetics (WT *versus* DNA-PK_cs_^−/−^). All HCT116-derived cell lines were cultured in Dulbecco’s modified Eagle’s medium containing 10% fetal calf serum (FCS) and 1% penicillin/streptomycin (p/s). SW48 human CRC cells were received from Dr. Maja Tomicic-Christmann (University Medical Center Mainz, Mainz, Germany) in 2014 and LS174T cells were a kind gift of Dr. Thomas Brunner (University of Konstanz, Konstanz, Germany) in 2015. Cells were re-authenticated by their p53 wild-type status and DNA damage-induced p53 stabilization. SW48 cells were grown in RPMI containing 10% FCS and 1% p/s. LS174T cells were maintained in IMDM medium (Pan Biotech, Aidenbach, Germany) with 10% FCS and 1% p/s. All cell lines were cultured at 37 °C in humidified atmosphere of 5% CO_2_ and 95% air. Cell culture medium and supplements were obtained from Gibco Life Technologies (Darmstadt, Germany) unless otherwise stated. Cell lines were mycoplasma negative as revealed by PCR detection and immunofluorescence microscopy with nuclear staining.

### Drugs and drug treatments

LA was dissolved in ethanol at a final stock concentration of 200 mM and added to medium at a final concentration of 1 mM. Cells were irradiated as indicated using a Gammacell 2000 (Nuklar, Frankfurt, Germany) equipped with a Cs^137^ source. Recombinant CDT holotoxin was added to the medium as indicated. The inhibitors (ATMi, AKT1/2i, AKT2i) were dissolved in DMSO providing a stock solution with 10 mM and added to the cells 1.5 h before treatment with CDT or *γ*-irradiation. For long-term incubations, that is, 72 h, cells were supplemented with the inhibitors once again after 24 h. The pan-caspase inhibitor Z-VAD-FMK was also dissolved in DMSO (20 mM stock) and added 20 h after CDT treatment or exposure to IR. Baf A1 was reconstituted in DMSO at a stock concentration of 10 mM and added 3 or 6 h prior to cell harvesting. CQ was dissolved in water (100 mM stock), diluted in medium and added 18 h prior to harvesting.

### Expression and purification of CDT

Recombinant CDT subunits (CdtA, CdtB, CdtC) from *Haemophilus ducyrei* were overexpressed as His-tagged fusion proteins in *E. coli BL21*. CDT subunits were isolated under denaturing conditions using Ni-NTA affinity chromatography and the holotoxin was reconstituted as described previously.^57^ In addition, a DNase-deficient CdtB mutant with a substitution in position 273 (D273R), the Mg^2+^-binding site, was used.^27^

### Transient transfection with siRNA against AKT1, ATG5 and p53

siRNA knockdown of AKT1, ATG5 and p53 was performed using siGENOME SMARTpool siRNA purchased from Dharmacon (Lafayette, CO, USA). Cells seeded in six-well plates were transfected at 40% confluency with 10 nM siRNA (p53, ATG5) or 20 nM siRNA (AKT1) using Lipofectamine RNAimax (Invitrogen, Darmstadt, Germany). Scrambled, non-sense siRNA (Dharmacon) was used as a negative control. Twenty-four hours following transfection, cells were treated with CDT or IR and harvested after different time points as indicated. AKT1, ATG5 and p53 knockdowns were monitored and verified by western blotting analysis.

### Annexin V/PI staining and flow cytometry

Cell death induction was analyzed by Annexin-V/PI staining as described previously.^59^ Briefly, cells grown in six-well plates were incubated with or without CDT for 48 h in the absence or presence of inhibitors. Attached and detached cells were harvested, washed with PBS and processed for Annexin-V/PI staining in Annexin-V-binding buffer (10 mM HEPES, pH 7.4, 140 mM NaCl, 2.5 mM CaCl_2_, 0.1% BSA) containing Annexin-V labeled with Alexa Fluor 488 (ratio 1 : 20; Miltenyi Biotech, Gladbach, Germany). After incubation on ice for 15 min, propidium iodide (0.5 g/ml) was added and cells were analyzed by flow cytometry using a FACS Canto II (BD Biosciences, Heidelberg, Germany). Analysis was performed as described using the FACSDiva software (BD Biosciences).

### Analysis of cell cycle distribution

Cell cycle distribution and subG1 population were assessed as previously described.^59^ Briefly, HCT116 cells were treated with increasing doses of CDT or exposed to IR. After 24 h, adherent cells were detached by trypsin incubation and pooled with cells floating in the cell culture supernatant. The cells were then harvested by centrifugation and washed twice in PBS. After ethanol precipitation at −20 °C overnight, the cell pellets were resuspended in PBS supplemented with RNase A (20 *μ*g/ml). Following incubation for 1 h at RT, PI was added to a final concentration of 10 *μ*g/ml and cells were analyzed for DNA content by flow cytometry using FACS Canto II (Becton Dickinson, Heidelberg, Germany). Cell cycle distribution and subG1 population were assessed with the FACS Diva software (Becton Dickinson).

### Confocal immunofluorescence microscopy

Cells grown on cover slips were treated with CDT or IR for different time points as indicated. Cells were further processed and stained as described.^23, 24^ Cells were fixed in 4% paraformaldehyde followed by ice-cold methanol at −20 °C and then blocked in PBS containing 5% (w/v) bovine serum albumin and 0.3% (v/v) Triton X-100 for 1 h. Samples were incubated with the respective primary antibody directed against *γ*-H2AX (no. ab81299; Abcam, Cambridge, UK), LC3B (no. 3868; Cell Signaling Technology, Denver, UK) and LAMP-1 (no. 9091; Cell Signaling Technology). Following several washing steps, samples were incubated with the secondary antibody labeled with AlexaFluor 488 (Life Technologies, Darmstadt, Germany) and DNA was counterstained with TO-PRO-3 (Life Technologies) for 15 min. Finally, cells were mounted using VectaShield (Vector Labs, Burlingame, CA, USA) and analyzed by confocal microscopy with a Zeiss Axio Observer.Z1 microscope equipped with a LSM710 laser-scanning unit (Zeiss, Oberkochen, Germany). Images were acquired by the ZEN software (Zeiss) and analyzed using the ImageJ software (NIH, Bethesda, MD, USA). To this end, the number of *γ*-H2AX-foci per nucleus was quantified (50–100 cells/treatment, *n*=3), while the intensity of LC3B and LAMP-1 was assessed per microscope section (8–10 sections, *n*=3).

In order to perform a LysoTracker/LC3B co-staining, LysoTracker reagent (Life Technologies) was added to the medium (100 nM) and cells were harvested after 45 min of incubation. Cells were then fixed, processed for the LC3B staining as described above and finally analyzed by confocal microscopy.

### Western blotting analysis

Cells were harvested and incubated for 15 min in lysis buffer containing 25 mM Tris-HCl pH 8.0, 2 mM EDTA, 150 mM NaCl, 1% NP-40, 0.5% Na-Desoxycholate and 0.1% SDS supplemented with cOmplete protease inhibitor cocktail (Roche Diagnostics, Mannheim, Germany), 1 mM Na_3_VO_4_, 2 mM NaF and 1 mM PMSF. Afterwards, cell lysates were sonicated on ice followed by centrifugation. The protein content of clarified extracts was determined using the Bradford assay. Equal amounts of protein samples were then separated by SDS-PAGE and transferred onto a nitrocellulose membrane (Perkin Elmer, Rodgau, Germany) using a wet-blot chamber (Bio-Rad, Munich, Germany) as described.^60^ After blocking in 5% (w/v) fat-free milk in Tris-buffered saline (TBS) with 0.1% (v/v) Tween 20 (TBS-T) for 1 h, incubation with the primary antibody was conducted. After washing the membrane with TBS-T, it was incubated with the appropriate secondary antibody coupled to horseradish peroxidase (Santa Cruz Biotechnology, Heidelberg, Germany) for 1 h at RT. After final washing steps, proteins were detected by enhanced chemiluminescence using Western Lightning Plus-ECL (Perkin Elmer, Erlangen, Germany).

The following primary antibodies were used: anti-heat shock protein (Hsp90) *α/β* (F8, mouse monoclonal; no. sc-13119), anti-p62 (mouse monoclonal; no. sc-28359) and anti-p53 (DO-1, mouse monoclonal; no. sc-126) (all from Santa Cruz Biotechnology), anti-*β*-Actin (mouse monoclonal, no. A2228, Sigma-Aldrich, Deisenhofen, Germany), anti-*γ*-H2AX (Ser139, rabbit monoclonal; no. ab81299; Abcam), anti-AKT (pan, rabbit monoclonal, no. 4691), anti-AKT1 (rabbit monoclonal, no. 2938 T), anti-phospho-AKT (Ser-473, rabbit monoclonal, no. 4060), anti-ATG5 (rabbit monoclonal, no. 12994), anti-LC3B (rabbit monoclonal; no. 3868), anti-LAMP-1 (rabbit monoclonal; no. 9091), anti-phospho-p53 (Ser15, rabbit polyclonal; no. 9284), anti-mTOR (rabbit monoclonal; no. 2983) and anti-phospho-mTOR (Ser2448, rabbit monoclonal; no. 5536), anti-phospho-p70 S6 kinase (Thr389, rabbit monoclonal; no. 9234), and anti-phospho-4EBP1 (Thr37/46, rabbit monoclonal; no. 2855) (all from Cell Signaling Technology).

### ICW analysis

ICW analysis was performed as previously described.^61^ Cells were seeded at 2 × 10^4^ cells/well and treated with increasing doses of CDT (5, 50, 500 ng/ml) or CDT^mut^ (500 ng/ml) in triplicates in 96-well plates. After 24 h, cells were washed with PBS and fixed with 4% paraformaldehyde for 10 min at RT. Upon washing with PBS, paraformaldehyde was neutralized using NH_4_Cl (20 mM). Next, cells were permeabilized with 0.2% Triton X-100 in PBS. Following washing with PST (PBS, 0.2% Triton X-100, 2% FCS), cells were blocked with MAXBlock Blocking Medium (Active Motif) supplemented with RNase A (Sigma) and cOmplete Protease Inhibitor (Roche) for 1 h at RT. Cells were then incubated with *γ*H2AX antibody (1 : 200 in PST) for 2 h at RT. As secondary antibody, an infrared fluorescent dye-conjugated antibody (1 : 1000 in PST; 800 nm; LiCor Biosciences, Bad Homburg, Germany) was used for 1 h at RT. For DNA labeling, RedDot2 (1 : 1000 in PST; Biotum, Fremont, CA, USA) was added to the secondary antibody solution. Measurements were performed using an Odyssey Infrared Imaging Scanner (Li-Cor Biosciences). DNA and *γ*-H2AX staining were simultaneously visualized. Statistical analysis in the Image Studio Light (Version 5.2; Li-Cor Biosciences) was performed to determine the *x*-fold increase in *γ*-H2AX signal normalized to the DNA content per well in relation to the control.

### Analysis of autophagy induction by flow cytometry

Autophagy induction was assessed using the CytoID Green Autophagy Detection Kit (Enzo Life Science, Lörrach, Germany). Cells were seeded in 6 cm dishes, treated with increasing CDT doses or exposed to IR as indicated and incubated for up to 72 h. Subsequently, both attached and detached cells were harvested, pelleted by centrifugation and washed with PBS. After staining of the cells according to the manufacturer’s protocol, they were analyzed by flow cytometry and CytoID fluorescence was determined using the BD FACSDiva software (BD Biosciences).

### Determination of cell viability by MTS assay

Cells grown in 96-well plates were incubated for 72 h with CDT in the absence or presence of inhibitors. Viability was determined using CellTiter 96 AQueous OneSolution Cell Proliferation Assay (Promega, Mannheim, Germany) according to the manufacturer’s instructions as previously described.^62^ Absorbance was measured with a 96-well plate reader (TECAN, Crailsheim, Germany).

### Statistical analysis

All experiments were performed independently three times, except otherwise stated. Results from representative experiments are shown. Values are displayed as means+S.E.M. using the GraphPad Prism 5.0 Software (GraphPad Software Inc., San Diego, CA, USA). Statistical analysis was performed using two-sided Student’s *t*-test and statistical significance was defined as *P*<0.05.
