# Supplementary material for: AKT2 suppresses pro-survival autophagy triggered by DNA double-strand breaks in colorectal cancer cells
Source: Cell Death Dis. 2017 Aug 24;8(8):e3019–. doi: 10.1038/cddis.2017.418 (PMC5596597; doi:10.1038/cddis.2017.418)
Supplement: Supplementary Figures [file cddis2017418x1.docx]

**Supplementary Information**

**Figure 1**

**
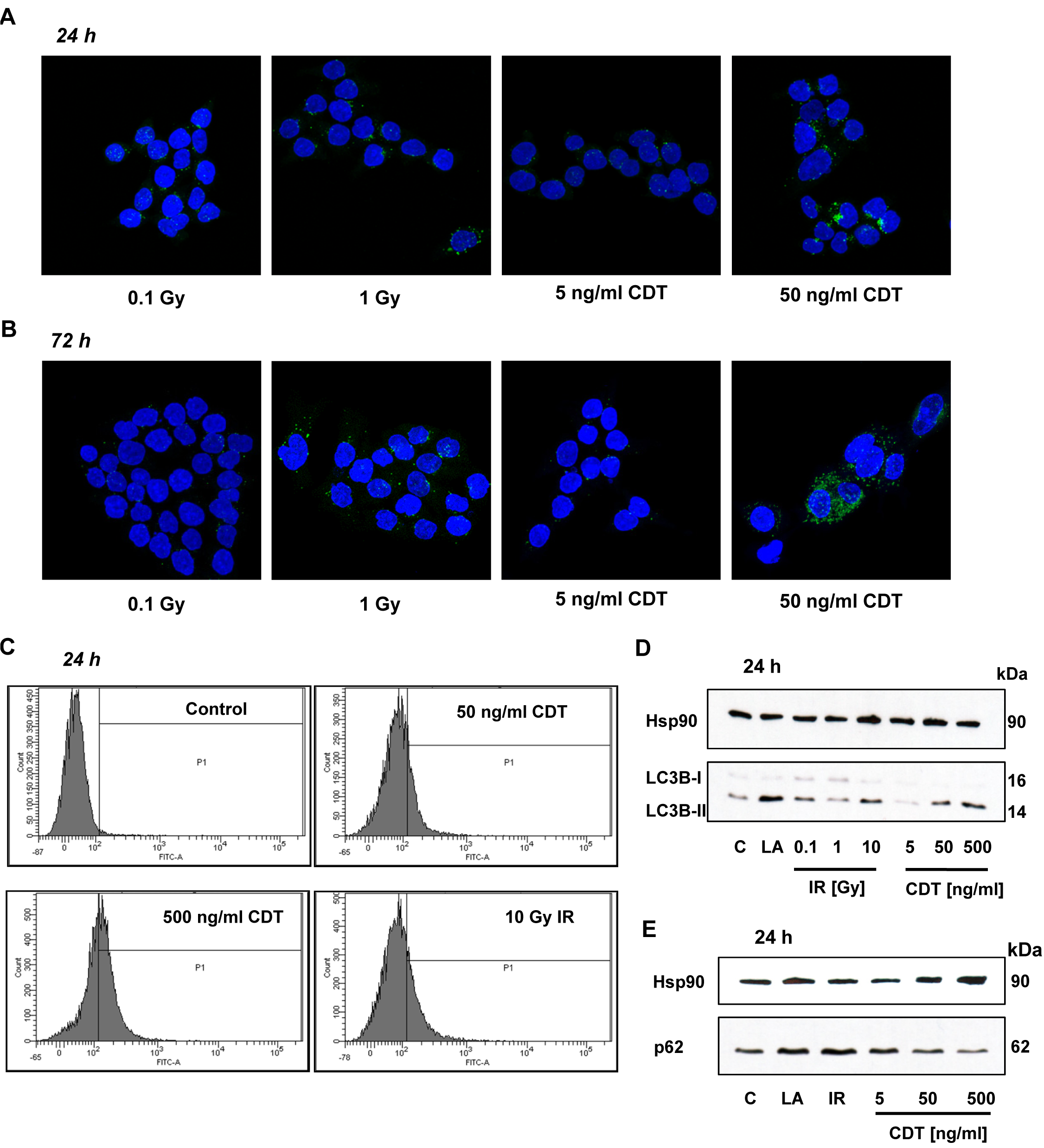
**

**Figure 1:** Dose-dependent induction of autophagy upon CDT and IR treatment. **(A)** and **(B)** Time- and dose-dependent induction of the autophagy marker LC3B. HCT116 cells were treated with CDT or exposed to γ-irradiation and incubated for 24 h (A) or 72 h (B). Cells were then fixed and stained for LC3B (green). Nuclei were counterstained by TO-PRO-3 (blue) and images were acquired by confocal microscopy. **(C)** Dose-dependent induction of autophagic vesicles after 24 h. HCT116 cells were treated as described and harvested after 24 h. Autophagic vesicles were stained with CytoID^®^ Autophagy detection kit and measured by flow cytometry. Representative histograms are shown. **(D)** and **(E)** Impact of CDT and IR on LC3B-II and p62 levels after 24 h. Cells were treated with CDT or IR in a dose-dependent manner and harvested after 24 h. Samples were analyzed by SDS-PAGE and Western Blotting as indicated. Hsp90 was visualized as loading control. α-lipoic acid (LA) was used as positive control.

**Figure 2**


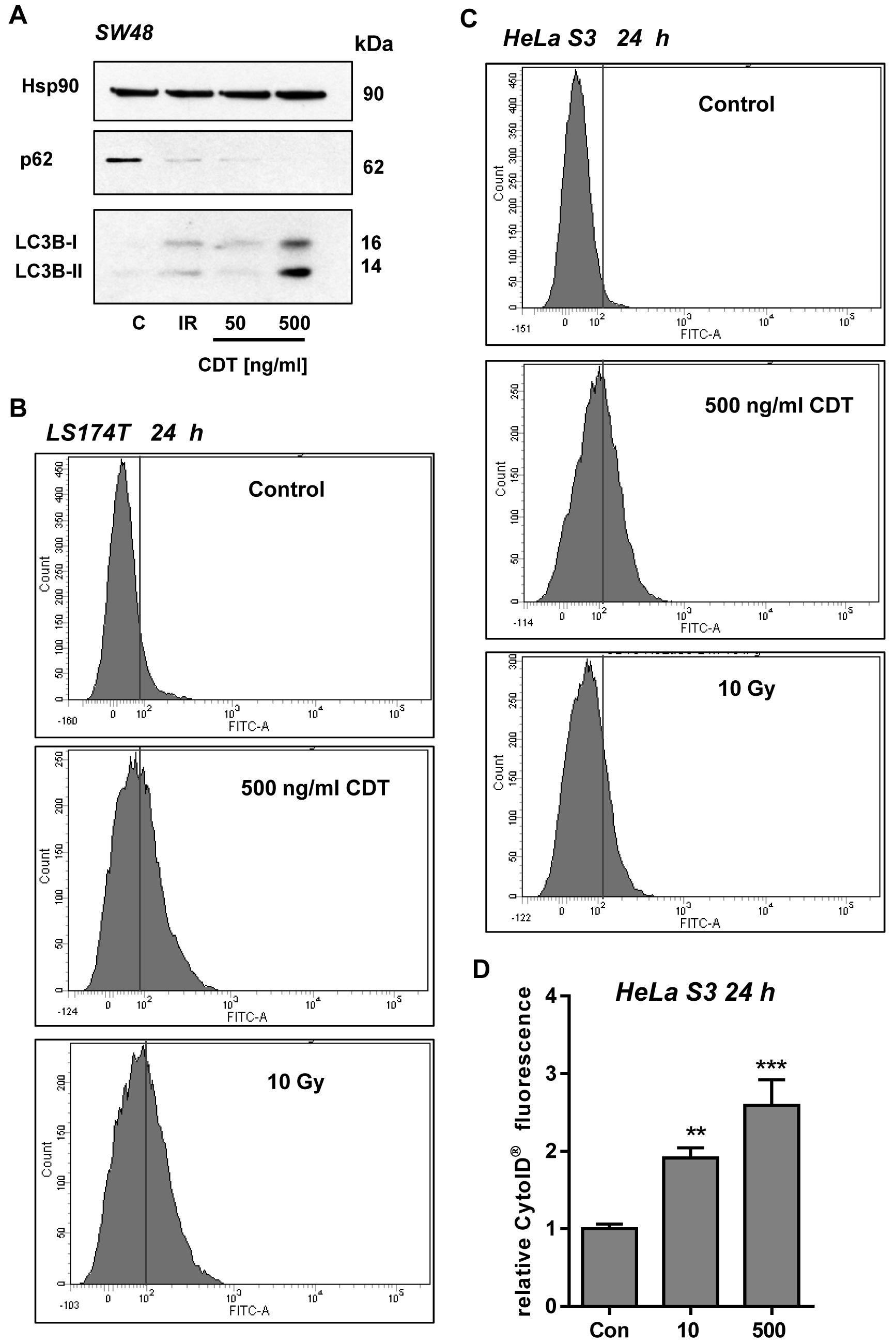


Figure 2: DSB-induced autophagy in SW48, LS174T and HeLa S3 cells. (A) Assessment of LC3B and p62 levels in SW48 colorectal cancer cells upon DNA damage. Cells were treated with CDT or exposed to IR. After 24 h, cells were harvested and samples were analyzed by SDS-PAGE and Western Blotting as indicated. Hsp90 was visualized as loading control. (B) LS174T colorectal cancer cells were exposed to CDT (500 ng/ml) or ionizing radiation (10 Gy). Cells were harvested after 24 h and autophagic vesicles were stained with CytoID^®^ Autophagy detection kit followed by flow cytometry. Representative histograms are shown. (C) and (D) Formation of autophagic vesicles in HeLa S3 cervix carcinoma cells 24 h upon CDT or IR exposure. Cells were treated and analyzed as described in A. Representative histograms are depicted. (n=4); **p<0.01, ***p<0.001.

**Figure 3**


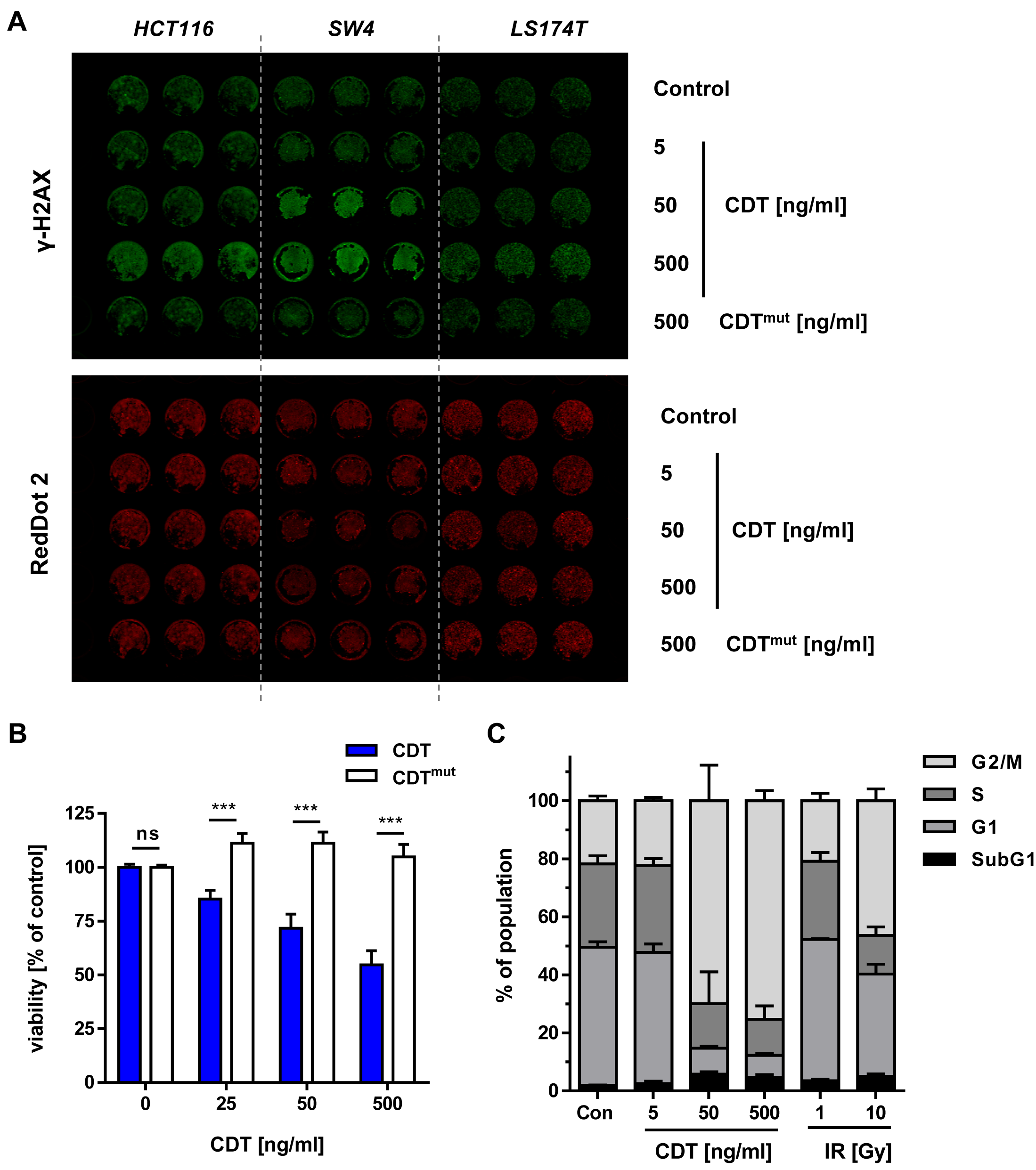


Figure 3: Impact of CDT and DNase I-defective CDT on cell viability. (A) HCT116, SW48 and LS174T CRC cells were treated with increasing doses of wildtype CDT (CDT^wt^) or DNase I-deficient CDT mutant (CDT^mut^) and incubated for 24 h. Cells were then processed for in-cell western (ICW) analysis. Following fixation and permeabilization, cells were stained for γ-H2AX and DNA was visualized using RedDot 2 dye. Signals were then measured in each well using an Odyssey Infrared Imaging Scanner. Shown are representative scans. (B) HCT116 cells were treated with increasing doses of wildtype CDT (CDT) or DNase I-deficient CDT mutant (CDT^mut^) and incubated for 72 h. Cell viability was then assessed by using MTS assay. (n=3); ns: not significant. ***p<0.001. (C) Impact of CDT and IR on cell cycle distribution and subG1 population. HCT116 cells were exposed to increasing doses of CDT or IR. Cells were harvested after 24 h, processed as described and analyzed by flow cytometry. (n=3)

**Figure 4**


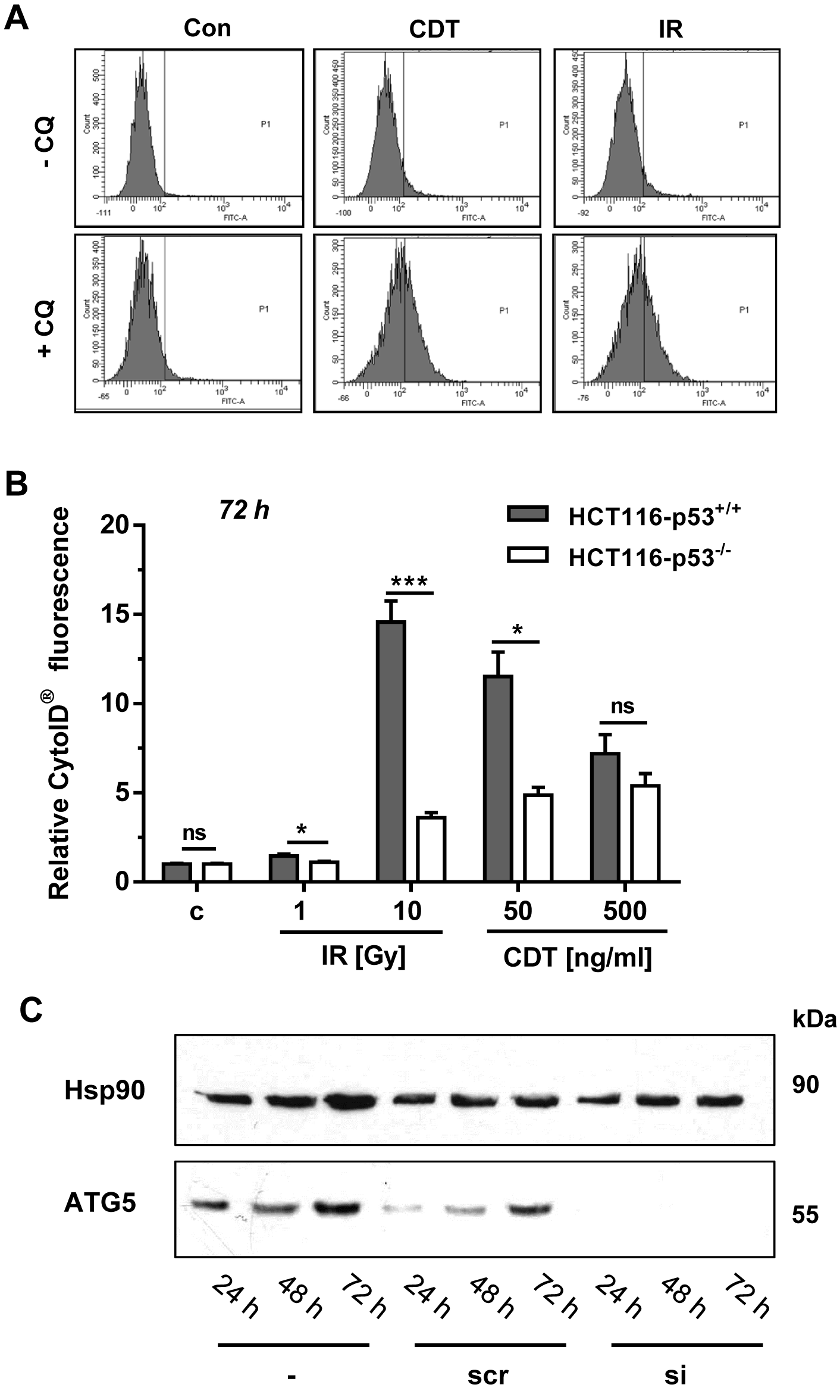


Figure 4: Autophagic flux and role of p53 in DSB-induced autophagy. (A) Formation of autophagic vesicles in the presence or absence of chloroquine (CQ). HCT116 cells were treated with CDT or exposed to IR. After 30 h, the autophagy inhibitor CQ and cells were further incubated for 18 h. Cells were harvested after 48 h and autophagy induction was measured using Cyto-ID^®^ Autophagy detection kit. (B) Determination of autophagic vesicles in HCT116-p53^+/+^ and HCT116-p53^-/-^ cells following DSB induction. Cells were irradiated or exposed to CDT as indicated and incubated for 24 h. Autophagosomes were stained with CytoID^®^ reagent and monitored by flow cytometry. (n=3); ns: not significant. *p<0.05, ***p<0.001. (C) siRNA-mediated knockdown of ATG5 in HCT116 cells. Cells were transfected with ATG5 siRNA or scrambled siRNA. Knockdown efficiency was monitored up to 72 h after transfection using Western blot analysis. Hsp90 was used as loading control.

**Figure 5**


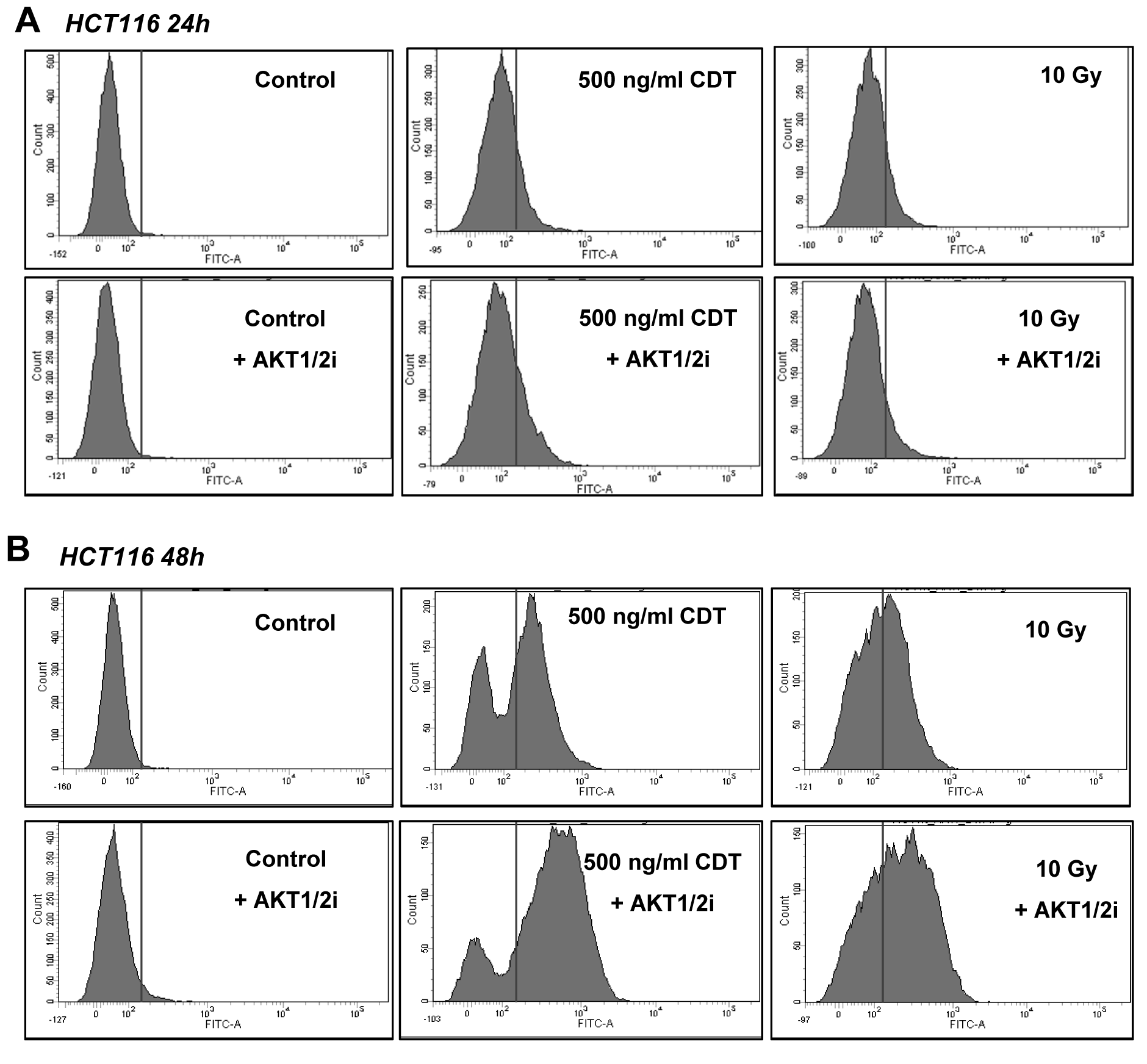


**Figure 5: Inhibition of AKT1/2 and DSB-induced formation of autophagosomes in HCT116 cells. (A)** and **(B)** Autophagosome formation. Cells were treated with CDT (500 ng/ml) or irradiated (10 Gy) in the absence or presence of an AKT1 and AKT2-specific inhibitor (AKT1/2i; 1 µM). Cells were harvested after 24 h **(A)** or 48 h **(B)** and then processed for CytoID^®^ staining followed by flow cytometry. Representative histograms are shown.

**Figure 6**

**
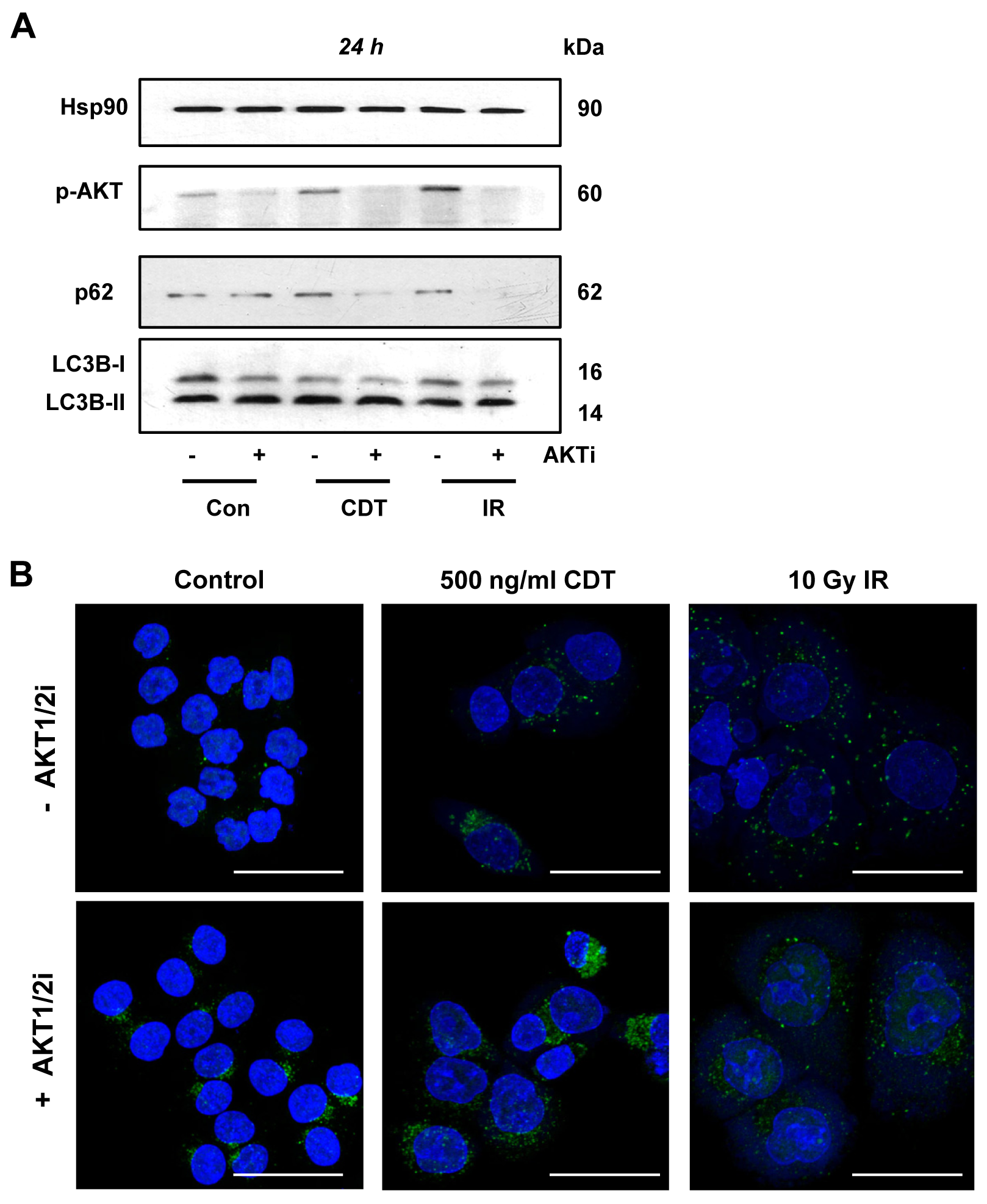
**

**Inhibition of AKT1/2 and DSB-induced formation of LC3B-positive autophagic vesicles in HCT116 cells. (A)** Effect of AKT on DSB-induced LC3B accumulation and p62 degradation. Cells were treated with CDT (500 ng/ml) or irradiated (10 Gy) in the absence or presence of an AKT1 and AKT2-specific inhibitor (AKT1/2i; 1 µM). After 24 h. the samples were subject to SDS-PAGE followed by western blot detection. Hsp90 served as loading control. **(B)** LC3B staining. Cells were treated as described above, processed for LC3B staining and analyzed by confocal immunofluorescence microscopy. Nuclei were counterstained with To-Pro-3. Representative pictures are shown. Size bar indicates 20 µm.

**Figure 7**

**
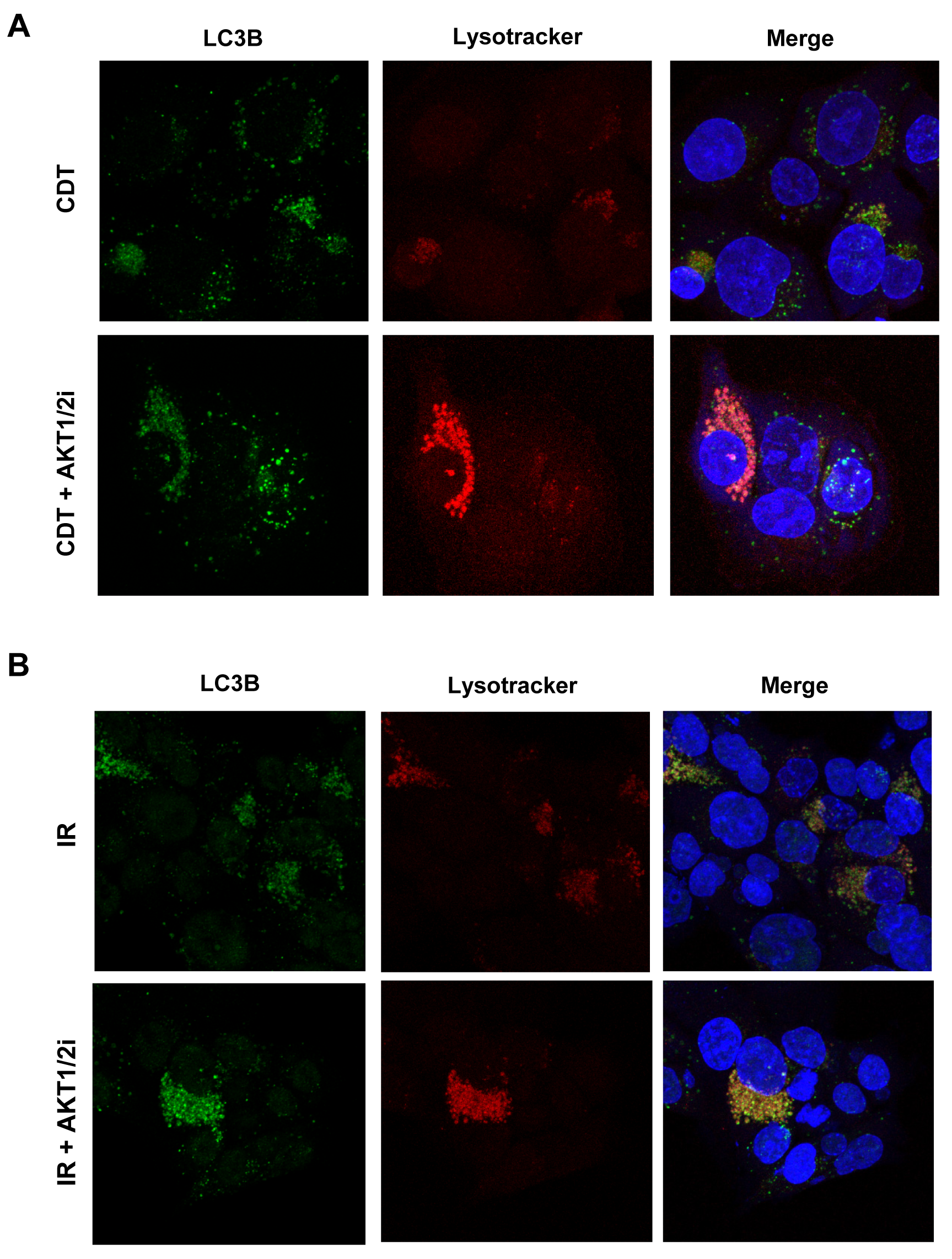
**

**Figure 7: Impact of AKT on DSB-induced autophagic flux (A**) and **(B)** HCT116 cells were exposed to CDT (500 ng/ml) or IR (10 Gy) and incubated for 48 h in the absence or presence of an AKT1 and AKT2-specific inhibitor (AKT1/2i; 1 µM). 45 min before the end of the experiment, LysoTracker® (100 nM) was added to the medium. Cells were then fixed and processed for LC3B staining as described. Lysotracker/LC3B co-staining was analyzed by confocal microscopy. Representative pictures are shown.

**Figure 8**


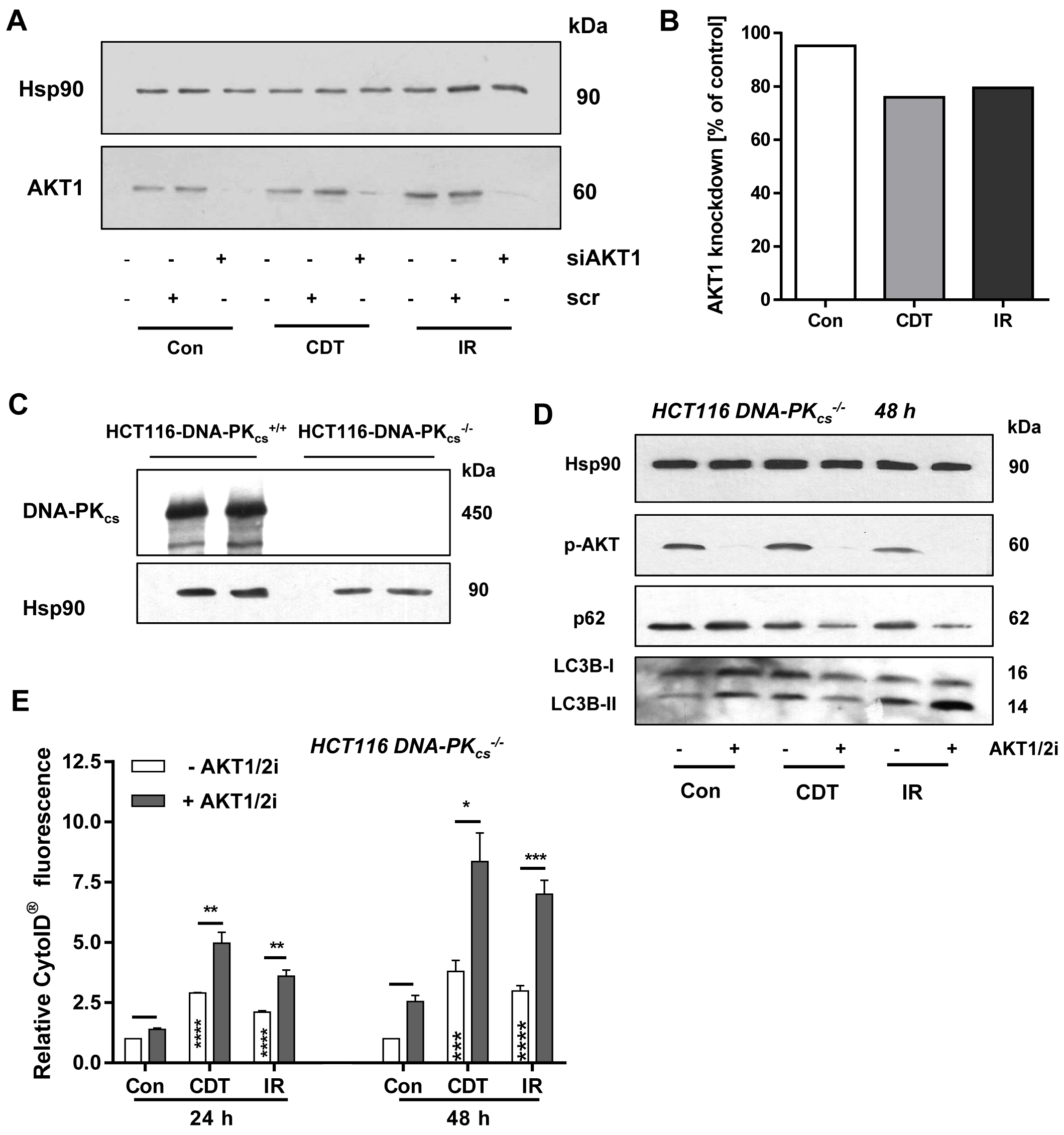


Figure 8: Impact of DNA-PK on CDT-induced autophagy and AKT (A) Transient siRNA-mediated knockdown of AKT1. HCT116 cells were transfected with AKT1 siRNA or scrambled siRNA. After 24 h, cells were exposed to CDT (500 ng/ml) or IR (10 Gy) and incubated for additional 48 h followed by western blot analysis using a AKT1-specific antibody. Hsp90 served as loading control. (B) Densitometric evaluation of AKT1 knockdown in HCT116 cells. (C) Analysis of DNA-PK_cs_ in HCT116 cells. DNA-PK_cs_-proficient and –deficient HCT116 cells were harvested and whole cell lysis was performed. Protein samples were then separated by SDS-PAGE followed by Western blot detection of DNA-PK_cs_. Hsp90 served as loading control. (D) HCT116-DNA-PK_cs_^-/-^ cells were exposed to CDT (500 ng/ml) or IR (10 Gy) with or without AKT1/2 inhibition. Cells were harvested after 48 h. Samples were analysed by SDS-PAGE and Western Blotting as indicated. (E) Determination of autophagic vesicles in HCT116-DNA-PK_cs_^-/-^ cells. Cells were treated as described above for 24 or 48 h, stained with CytoID^®^ Autophagy detection kit and measured by flow cytometry (n=3). ns: not significant; ****p<0.0001, ***p<0.001, **p<0.01, *p<0.05.
